# *COL7A1* indicates crucial potential as a basal membrane-related prognostic biomarker and therapeutic target in lung adenocarcinoma

**DOI:** 10.3389/fphar.2025.1543193

**Published:** 2025-02-14

**Authors:** Jiao Zhang, Rui Zi, Ping Hu, Zongying Jiang, Ye Lv, Haixia Zhang, Yanjiao Zhao, Yan Wang, Lujun Zhao

**Affiliations:** ^1^ Department of Radiation Oncology, Tianjin Medical University Cancer Institute and Hospital, Tianjin, China; ^2^ National Clinical Research Center for Cancer, Tianjin Medical University Cancer Institute and Hospital, Tianjin, China; ^3^ Tianjin’s Clinical Research Center for Cancer, Tianjin, China; ^4^ Key Laboratory of Cancer Prevention and Therapy, Tianjin, China; ^5^ The Third Department of Medical Oncology, General Hospital of Ningxia Medical University, Yinchuan, China; ^6^ The First Department of Medical Oncology, General Hospital of Ningxia Medical University, Yinchuan, China; ^7^ Department of Pathology, General Hospital of Ningxia Medical University, Yinchuan, China

**Keywords:** lung adenocarcinoma, basal membrane, *COL7A1*, prognosis, immune cell infiltration, immunotherapy

## Abstract

**Introduction:**

Lung adenocarcinoma (LUAD) is the most prevalent subtype of lung cancer. Basal membrane (BM) is important to the invasive processes of LUAD. Our object is to explore hub BM-related genes in LUAD.

**Methods:**

The gene expression data of LUAD were downloaded from The Cancer Genome Atlas and Gene Expression Omnibus databases. The weighted gene co-expression network analysis and differentially expressed gene analysis were used to identify candidates. Gene Ontology and Kyoto Encyclopedia of Genes and Genomes enrichment analyses were used to evaluate their functions. Univariate Cox regression analysis was used to evaluate the prognostic value, and multivariate Cox regression analysis was used to verify its independence as a prognostic risk factor. The qPCR and Western blot were performed to ascertain the hub gene expression. The survival curve of two groups was drawn using Kaplan-Meier method. The hub gene-related immune characteristics were analyzed in independent cohorts by ESTIMATE and CIBERSORT methods.

**Results:**

We successfully identified *COL7A1* as a BM-related prognostic biomarker in LUAD, with elevated expression compared to controls, and associated with poor prognosis. Functional enrichment analysis revealed it was involved in pathways related to cell proliferation and inflammation like ECM-receptor interaction. Time-dependent ROC analysis results showed that the AUC of *COL7A1* in predicting 1-, 3-, and 5-year survival all exceeded 0.78. Immune infiltration characteristic analysis showed that the higher *COL7A1* expression group exhibited lower ESTIMATE scores and higher TIDE scores.

**Discussion:**

Our study identified *COL7A1* as a reliable BM-related prognostic biomarker, providing a new reference for the mechanistic understanding and target therapy of LUAD.

## 1 Introduction

Lung adenocarcinoma (LUAD) constitutes the most prevalent histological subtype among Non-small-cell lung cancer (NSCLC) cases, comprising nearly half (47%) of all occurrences (Gridelli et al., 2015). This subtype arises from alveolar cells and bronchiolar epithelial cells (Herbst et al., 2018). Currently, the 5-year survival rate for LUAD patients stands below 50%, while those with distant metastases are only 7% (Hao et al., 2022). Current molecular targeted therapy offers novel immunotherapy options for LUAD treatment (Shao et al., 2021; Yang et al., 2022), providing significant benefits to specific subgroups of patients (Saab et al., 2020). However, the progress of LUAD treatment has been slow, and most patients develop resistance to clinical therapies (Fan and Mao, 2017). Therefore, the identification of novel biomarkers that can serve as therapeutic targets for LUAD patients is imperative. This is necessary to effectively evaluate the prognosis of patients and provide the foundation for individualized LUAD diagnosis and treatment.

Basal membrane (BM), a specialized extracellular matrix (ECM) generated by epithelial and endothelial cells (Yurchenco, 2011), holds a central position in the growth and operation of various tissues (Chang and Chaudhuri, 2019). It is crucial for maintaining tissue integrity, cell signaling, and barrier functions (Mukwaya et al., 2021). Notably, BM performs the role of a natural barrier, preventing the invasion of primary tumor cells into neighboring stromal tissue (Kelley et al., 2014). As cancers become aggressive, they must penetrate the BM to escape, ultimately leading to metastasis which is a major cause of cancer-related deaths (Reuten et al., 2021). BM has been identified as a key player in the invasive processes of various cancer types, including oral cancer (Wilson et al., 1999) and breast cancer (Wisdom et al., 2020). Consequently, understanding the underlying mechanisms of BM in LUAD is of utmost importance. Recent studies have revealed that the risk score models constructed using BM-related genes can significantly estimate the prognosis and immunologic therapeutic responsiveness in LUAD patients. For instance, Shi et al. have developed a model based on four genes, including *HMCN2*, *FBLN5*, *ADAMTS15*, and *LAD1,* in LUAD patients, effectively predicting prognosis and immunotherapy response (Shi et al., 2023). Furthermore, Zhang et al. have suggested that *NELL2* may be a potential modulator of the BM-related immune system in LUAD (Zhang et al., 2023). However, these studies have involved a substantial number of genes in constructing their models, limiting their clinical applicability. Therefore, a more in-depth analysis of BM-related genes in LUAD is necessary to enhance the overall prognostic accuracy.

In this study, our goal is to delve into a novel BM-related biomarker linked to the prognosis of LUAD and to reveal its functional significance and prognostic impact in patients. Our findings are expected to contribute novel insights that can inform immunotherapy strategies tailored to LUAD patients.

## 2 Materials and methods

### 2.1 Subjects

The RNA-seq data and clinical information of 544 LUAD (486 LUAD samples and 58 control samples) were downloaded from The Cancer Genome Atlas (TCGA, https://tcga-data.nci.nih.gov/tcga/) database.

In addition, more LUAD related datasets were downloaded from the Gene Expression Omnibus (GEO) database (https://www.ncbi.nlm.nih.gov/geo/), including GSE115002 (52 LUAD samples and 52 controls), GSE10072 (58 LUAD samples and 49 controls), GSE72094 (386 LUAD samples with pathology and survival information), and GSE68465 (301 LUAD samples with pathology and survival information).

### 2.2 Weighted gene co-expression network analysis

The weighted gene co-expression network analysis (WGCNA) was performed using the R package “WGCNA” (version 1.72-5) (Langfelder and Horvath, 2008) to identify modules that exhibited a significant association with the LUAD.

### 2.3 Differentially expressed gene analysis

The differentially expressed genes (DEGs) were screened using “limma” package of R (version 3.56.2) (Ritchie et al., 2015) with the cut-off criteria of |log_2_FC| >0.5 and p. adjust <0.05.

### 2.4 Functional enrichment analysis

The Gene Ontology (GO), Kyoto Encyclopedia of Genes and Genomes (KEGG) pathway enrichment analysis, and Gene Set Enrichment Analysis (GSEA) were conducted using the “clusterProfiler” R package (version 4.8.3) (Yu et al., 2012). The “DOSE” R package (version 3.26.1) was used to perform disease ontology (DO) analysis (Yu et al., 2015). A p-value <0.05 was considered statistically significant.

### 2.5 Survival analysis

The R packages “survival” were performed to estimate the overall survival (OS) of patients in different groups. The survival curve of two groups was drawn using Kaplan-Meier method, and the significance of the survival difference was determined with log-rank test. Univariate Cox regression analysis was utilized to identify prognostic risk factors. Multivariate Cox regression analysis was used to determine whether the biomarker is an independent prognostic factor.

### 2.6 Immune cell infiltration analysis

The “CICERSORT” was utilized to characterize the composition of immune cells for different groups (Newman et al., 2015). The proportions of a total of 22 immune cells were calculated for each sample. The immunityscore was calculated using the “estimate” function. Tumor immune dysfunction and exclusion (TIDE, http://tide.dfci.harvard.edu/) was utilized to assess the effect of immunotherapy across diverse groups, reflecting the tumor’s potential immune escape capabilities. A higher TIDE score was associated with a poorer response to immunotherapy. In addition, Pearson correlation analysis was performed to assess the association between immune checkpoint genes’ expression levels and the biomarker’s expression level.

### 2.7 Drug sensitivity analysis

Drug sensitivity data were collected from Genomics of Drug Sensitivity in Cancer (GDSC, http://www.cancerrxgene.org/). The IC50 values of drugs were calculated using R package “oncoPredict” (version 0.2) (Maeser et al., 2021).

### 2.8 Cell culturing

The normal human lung epithelial cells BEAS-2B (CL0044, Hunan Fenghui Biotechnology Co., Ltd., Hunan, China) and the human LUAD cells H1650 (BNCC100260, BeNa Culture Collection Co., Ltd., Henan, China), H1975 (CL0232, Hunan Fenghui Biotechnology Co., Ltd., Hunan, China), and H838 (BNCC100696, BeNa Culture Collection Co., Ltd., Henan, China) were obtained. All cell lines were maintained in the complete medium with the addition of 90% medium, 10% fetal bovine serum (FBS) (164210, Wuhan Pricella Biotechnology Co., Ltd., Wuhan, China), and 1% penicillin-streptomycin (P/S) solution (PB180120, Wuhan Pricella Biotechnology Co., Ltd., Wuhan, China). And all culture mediums were at 37°C in a humidified incubator containing 5% CO_2_.

### 2.9 Quantitative real-time PCR (qPCR)

The total RNA was extracted from cells by TriQuick total RNA Extraction Reagent (R1100, Beijing Solarbio ScienceandTechnology Co.,Ltd., Beijing, China) and reverse transcribed into cDNA. Next, qPCR was performed using the 2×RealStar Power SYBR qPCR Mix (GeneStar). The following thermocycling conditions were used for qPCR: 1 cycle at 95°C for 30 s (initial denaturation), followed by 40 cycles of 5 s at 95°C and 30 s at 60°C. GAPDH was the reference gene and the primer sequences were as follows: COL7A1 forward (5′-GGT​GTT​CCT​ACC​ACA​TGC​CA-3′) and COL7A1 reverse (5′-CCA​AGG​TCA​TGG​GAG​CCA​TT-3′); GAPDH forward (5′-GAA​GGT​GAA​GGT​CGG​AGT​C-3′) and GAPDH reverse (5′-GAA​GAT​GGT​GAT​GGG​ATT​TC-3′). The Ct value of each PCR reaction was readed using Stepone software and the relative expression level was normalized to GAPDH and calculated using $2^{-∆∆Ct}$ method (Livak and Schmittgen, 2001).

### 2.10 Western blot (WB)

Total protein from LUAD cells were extracted using radioimmunoprecipitation assay buffer (RIPA) Lysis Buffer (Beijing Solarbio ScienceandTechnology Co.,Ltd., Beijing, China). Following the protocol in previous study, WB was performed (Wang et al., 2016). In this experiment, the primary antibody was COL7A1 Polyclonal Antibody (19799-1-AP, Proteintech Group, Wuhan, China) and GAPDH Antibody (60004-1-Ig, Proteintech Group, Wuhan, China), and the secondary antibody was horseradase labeling of goat with anti-rabbit IgG 1:1,000 (ZB-2301-0.1mL, Beijing Zhong Shan-Golden Bridge Biological Technology Co., Ltd., Beijing, China) and horseradase labeling of goat anti-mouse IgG 1:1,000 (ZB-2301-0.1mL, Beijing Zhong Shan-Golden Bridge Biological Technology Co., Ltd., Beijing, China). Protein bands were visualized by fully automated chemiluminescence image analysis system. Finally, band intensities were analyzed using the ImageJ software.

### 2.11 Statistical analysis

The Wilcox test was conducted to determine if there is a significant difference in continuous variables between the two groups. As for correlation analysis, Shapiro-Wilk test was first conducted to determine whether the data conformed to normal distribution. Then, the data with normal distribution was subjected to a pearson correlation analysis, while the non-normal data was analyzed with spearman correlation analysis. The Pearson correlation was performed using R function “cor”. Statistical significance was considered at p < 0.05. All statistical analyses were conducted using R software (version 4.3.2).

## 3 Results

### 3.1 Identification of LUAD-related modules using WGCNA

**FIGURE 1:**
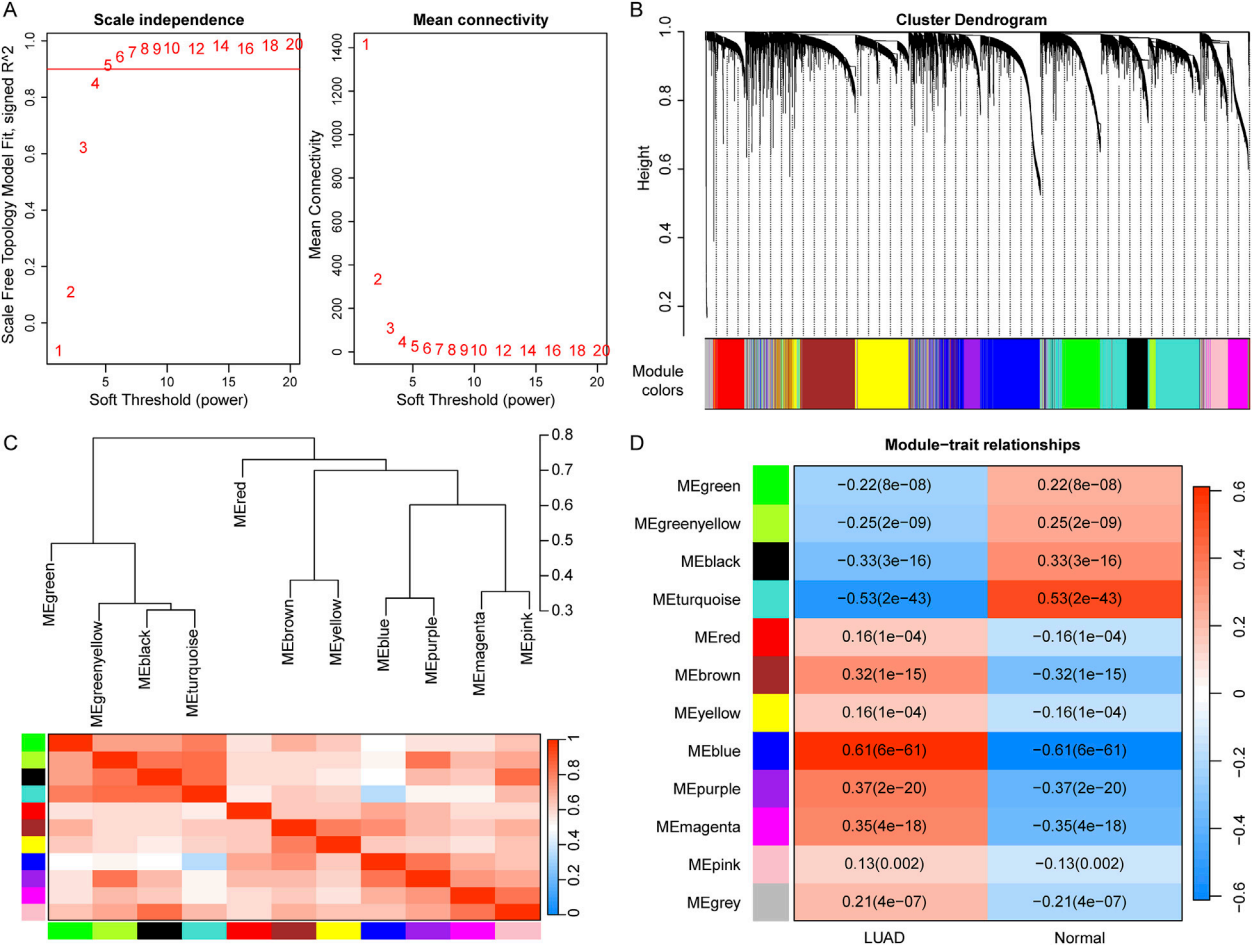
Identification of LUAD-related modules using WGCNA. **(A)** Determination of the soft-thresholding (power threshold β = 5). **(B)** Cluster dendrogram of gene modules. Each color represents a module, and the gray represents the gene set that cannot be aggregated to other modules. **(C)** Heatmap of eigengene adjacency. **(D)** Heatmap of the association of gene modules with LUAD and normal. Numbers in each cell indicate correlation and significance.

To identify potential modules associated with LUAD, we performed WGCNA on the TCGA dataset. The scale-free fit index signed *R* ^2^ > 0.9 and soft power of β = 5 were soft-thresholding parameters to construct the gene network (Figure 1A). As a result, we identified a total of 12 gene modules (Figure 1B). Then, the correlation between each gene module and the two groups including LUAD and control were calculated (Figures 1C,D). The results showed that nine modules, including “green”, “black”, “brown”, “greenyellow”, “purple”, “magenta”, “blue”, “turquoise”, and “grey”, were significantly correlated with LUAD (|Cor| > 0.2, p < 0.05). Therefore, the genes in these nine modules were selected for downstream analysis, containing a total of 871 genes (LUAD-related-genes) (Supplementary Table S1).

### 3.2 Screening the BM-related candidate genes in LUAD

**FIGURE 2:**
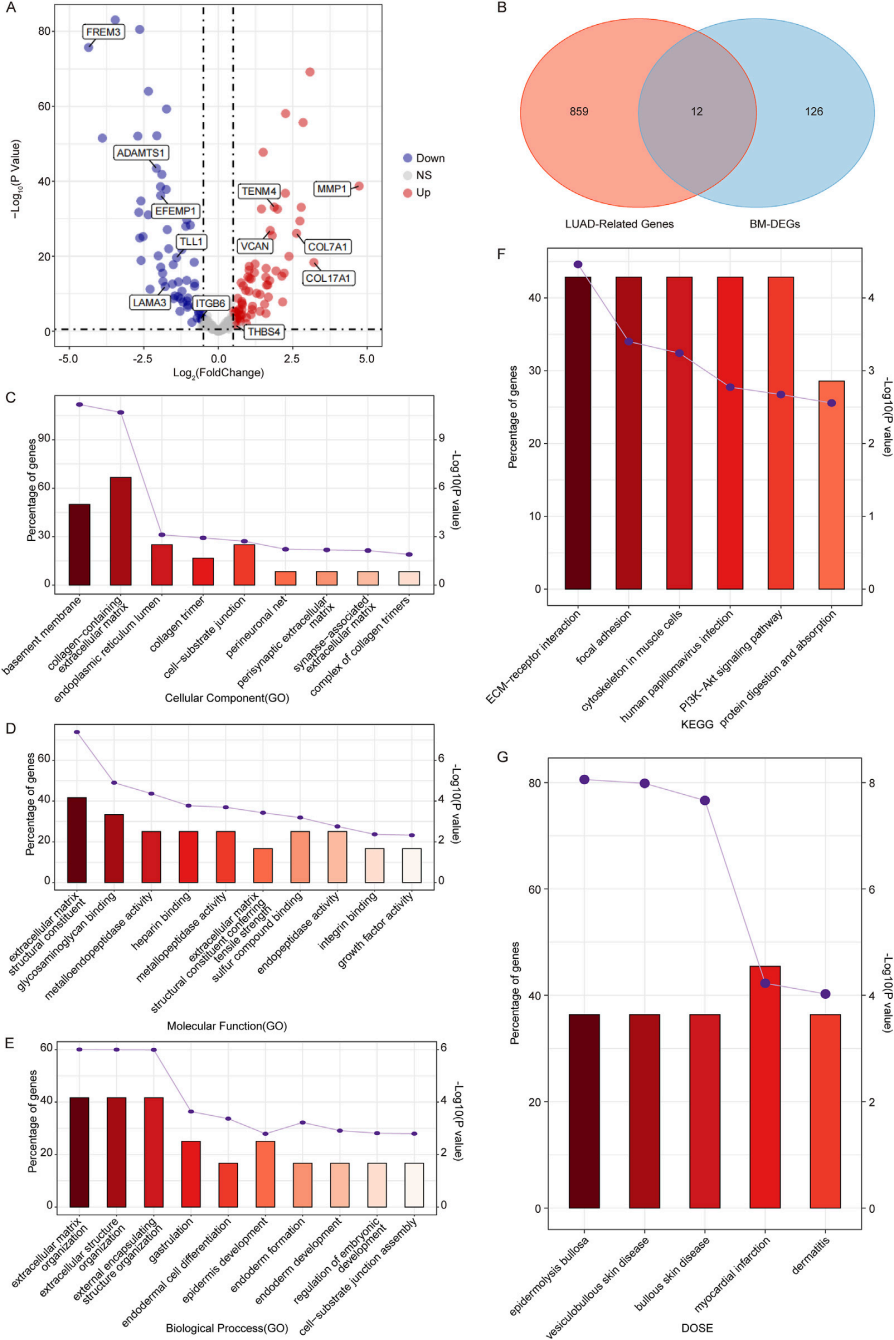
Identification of candidate genes associated with BM in LUAD. **(A)** Volcano plots of BM-related DEGs between LUAD samples and controls in the TCGA dataset. **(B)** Venn diagram of the WGCNA module genes and BM-DEGs. **(C, E)** The GO terms including CC **(C)**, MF **(D)**, and BP **(E)** of candidate genes. **(F, G)** The KEGG pathway enrichment **(F)** and DO enrichment **(G)** of candidate genes. (If more than 10 pathways, only the top 10 pathways with the smallest p-value were shown.)

To identify BM-related genes in LUAD, firstly, we downloaded 222 BM-related human proteins from a published study (Jayadev et al., 2022). Then we performed a DEG analysis between LUAD samples and controls in the TCGA dataset to screen BM-related DEGs (BM-DEGs). As a result, a total of 138 BM-DEGs were identified in the LUAD group compared to controls, including 65 upregulated genes and 73 downregulated genes (Figure 2A; Supplementary Table S2). Next, 12 overlapping genes, including *FREM3*, *DAMTS1*, *MMP1*, *EFEMP1*, *TENM4*, *VCAN*, *COL7A1*, *TLL1*, *COL17A1*, *LAMA3*, *ITGB6*, and *THBS4*, were identified by taking the intersection of the 871 LUAD-related-genes and 138 BM-DEGs (Figure 2B).

Going further, we performed GO and KEGG pathway enrichment analyses to determine the potential functions of the 12 candidates. The GO enrichment analysis showed that they were involved in nine CC terms such as basement membrane and collagen-containing extracellular matrix (Figure 2C); 16 MF terms like extracellular matrix structural constituent (Figure 2D); and 13 BP terms such as extracellular structure organization (Figure 2E). The KEGG enrichment analysis showed that the 12 candidates were significantly enriched in six pathways such as ECM-receptor interaction, focal adhesion, and PI3K-Akt signaling pathway (Figure 2F), which played important roles in cell apoptosis, proliferation, and migration.

In addition, we conducted a DO analysis on the 12 candidates, and we found that they were significantly enriched in five DO terms including epidermolysis bullosa, vesiculobullous skin disease, bullous skin disease, myocardial infarction and dermatitis (Figure 2G), suggesting that the BM-related candidate genes were significantly associated with these diseases. The detailed enrichment analysis results of the 12 candidates were shown in Supplementary Table S3.

### 3.3 Identification of *COL7A1* as a key BM-related biomarker in LUAD

**FIGURE 3:**
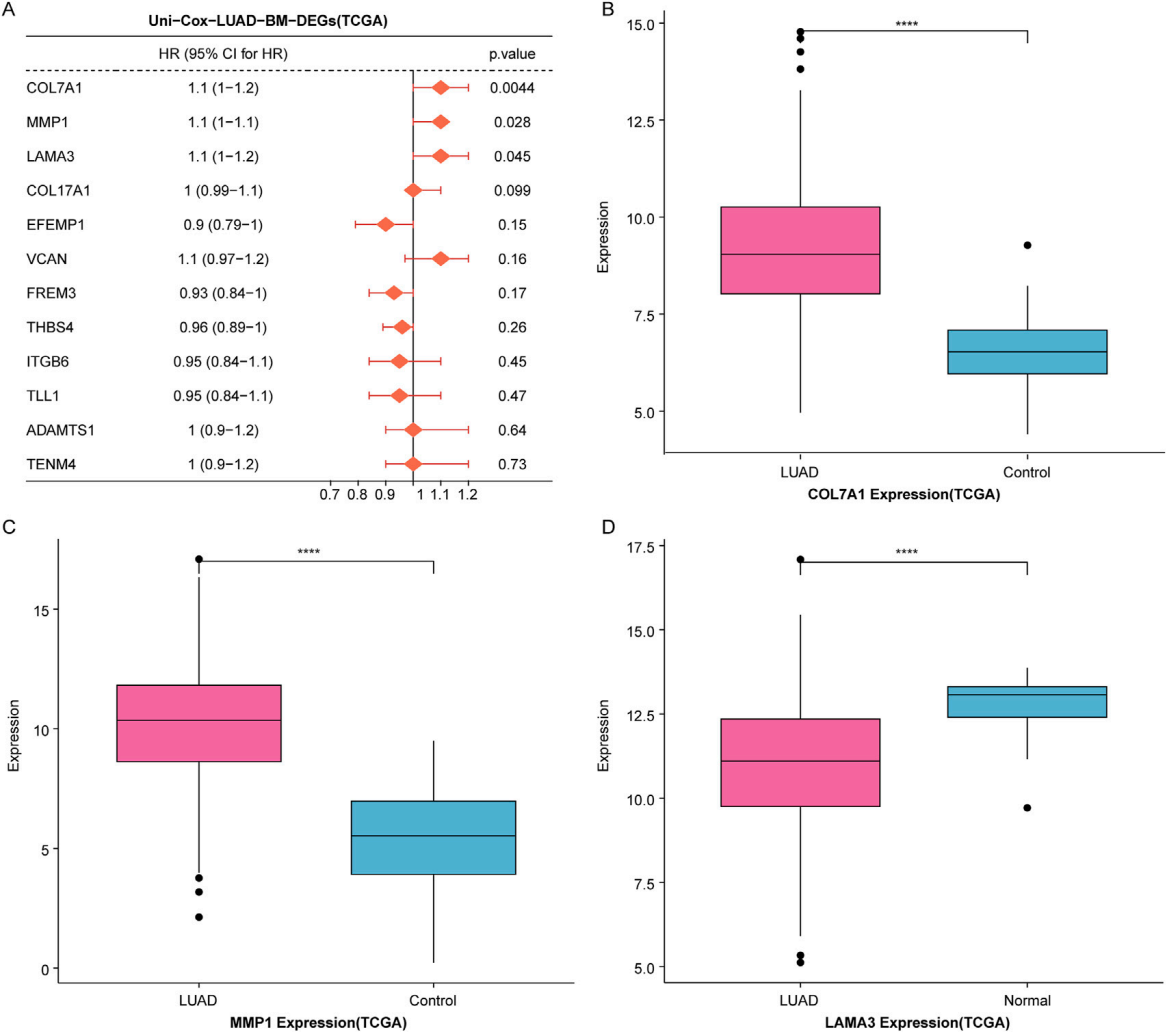
Identification of COL7A1 as a BM-related key biomarker in LUAD. **(A)** Univariate Cox regression analysis of 12 candidate genes. **(B–D)** The expression levels of three prognosis-associated genes, including *COL7A1* **(B)**, *MMP1* **(C)**, and *LAMA3* **(D)**, in LUAD samples and controls of the TCGA dataset (*p < 0.05, **p < 0.01, ***p < 0.001).

To further identify prognostic markers for LUAD patients, we performed univariate Cox regression analysis on the 12 candidate genes. The results showed a significant association between *MMP1*, *COL7A1*, *LAMA3* and LUAD prognosis (HR > 1 and p < 0.05) (Figure 3A). Subsequently, we observed the expression differences of *MMP1*, *COL7A1*, and *LAMA3* betweem LUAD samples and controls in the TCGA dataset, and we found that the expression of *COL7A1* and *MMP1* was significantly upregulated in LUAD samples compared to controls (Figures 3B,C), while *LAMA3* was significantly downregulated in LUAD samples (Figure 3D).

Studies had shown that *MMP1* was a key gene related to the prognosis of LUAD (Wang et al., 2024). *LAMA3* was a hub gene linked to LUAD drug resistance and was significantly associated with patient prognosis (Yu et al., 2023). It is reported that *COL7A1* encodes collagen type VII, which is a component of BM. However, the prognostic value of *COL7A1* in LUAD has not been investigated; therefore, *COL7A1* was chosen as a key BM-related gene for downstream analysis in LUAD.

### 3.4 Significant high expression of *COL7A1* in LUAD samples compared to controls

**FIGURE 4:**
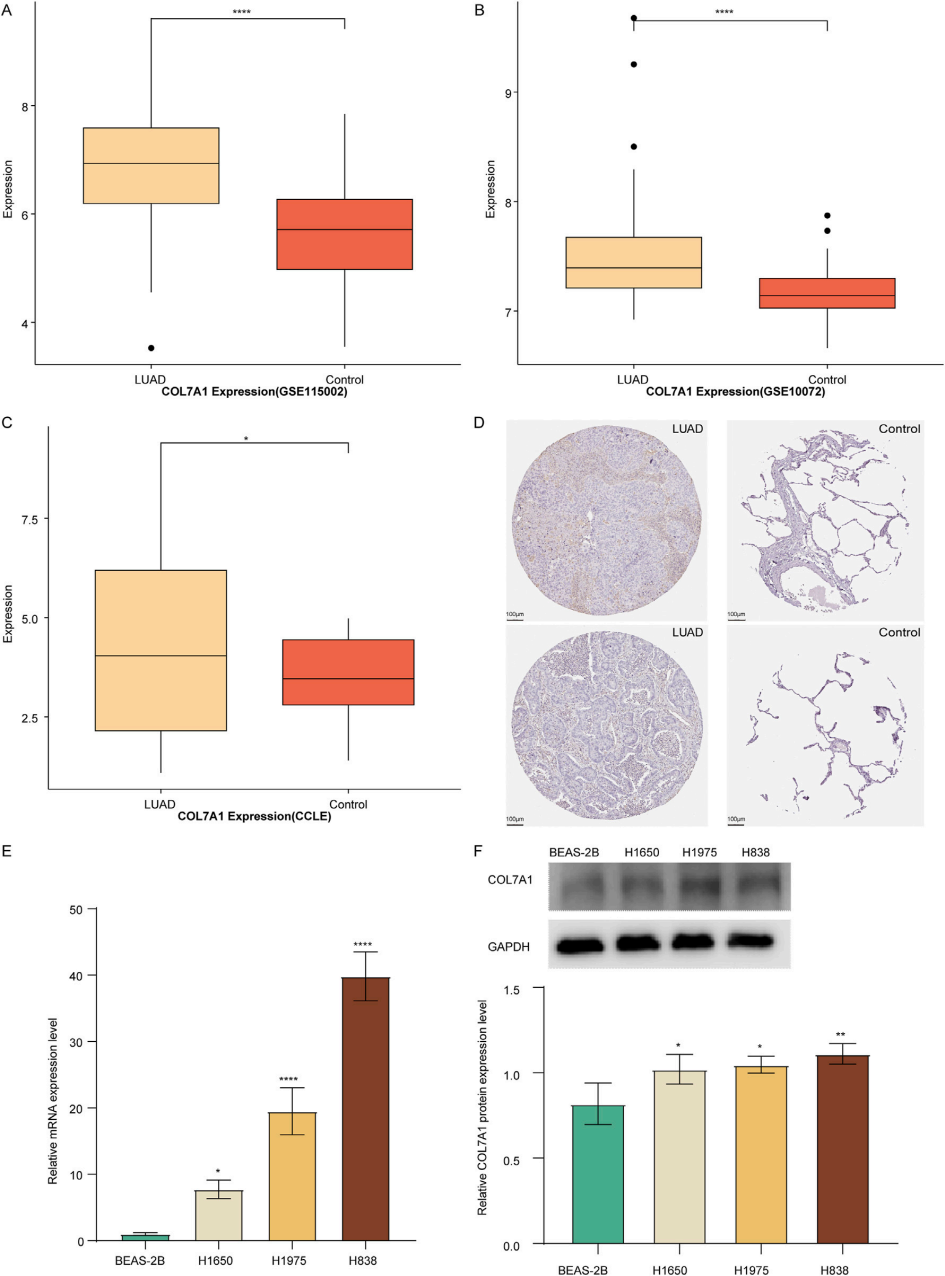
Validation of the *COL7A1* expression levels. **(A–C)** Box plot of *COL7A1* expression in LUAD and controls of GSE115002 **(A)**, GSE11072 **(B)** cohorts, and CCLE database **(C)**. **(D)** Immunohistochemical plots of *COL7A1* in lung tissue downloaded from the HPA database, with LUAD tissue on the left and normal lung tissue on the right. **(E, F)** Expression of *COL7A1* in LUAD determined by qPCR **(E)** and Western blot **(F)**. (*p < 0.05, **p < 0.01, ***p < 0.001).

To assess the role of *COL7A1* in LUAD, we observed its expression differences between LUAD and control samples. The results showed that in the GSE115002 and GSE11072 datasets, COL7A1 was significantly overexpressed in LUAD sample compared to controls (Figures 4A, B). Furthermore, validation using the CCLE database for LUAD cell lines supported our findings, demonstrating a significantly higher COL7A1 expression in LUAD cell lines compared to normal cell lines (Figure 4C). In addition, we observed the immunohistochemical maps of *COL7A1* in LUAD samples and controls based on the Human Protein Atlas (HPA) database and found that *COL7A1* showed higher expression in LUAD samples (Figure 4D). The results of qPCR and WB showed that both mRNA and protein levels of *COL7A1* were significantly upregulated in LUAD cells H1650, H19753, and H838 compared to normal human lung epithelial cells BEAS-2B (Figures 4E, F).

Going further, to determine the effect of *COL7A1* in the growth of LUAD, we employed the TCGA dataset to comprehensively analyze the relationship of its expression patterns with gender, age, clinical stage, grade, and TNM stage. Unfortunately, we did not observe significant differences in the expression of *COL7A1* among the different groups (Supplementary Figures S1A–F).

### 3.5 High *COL7A1* expression significantly associated with worse survival outcomes in LUAD

**FIGURE 5:**
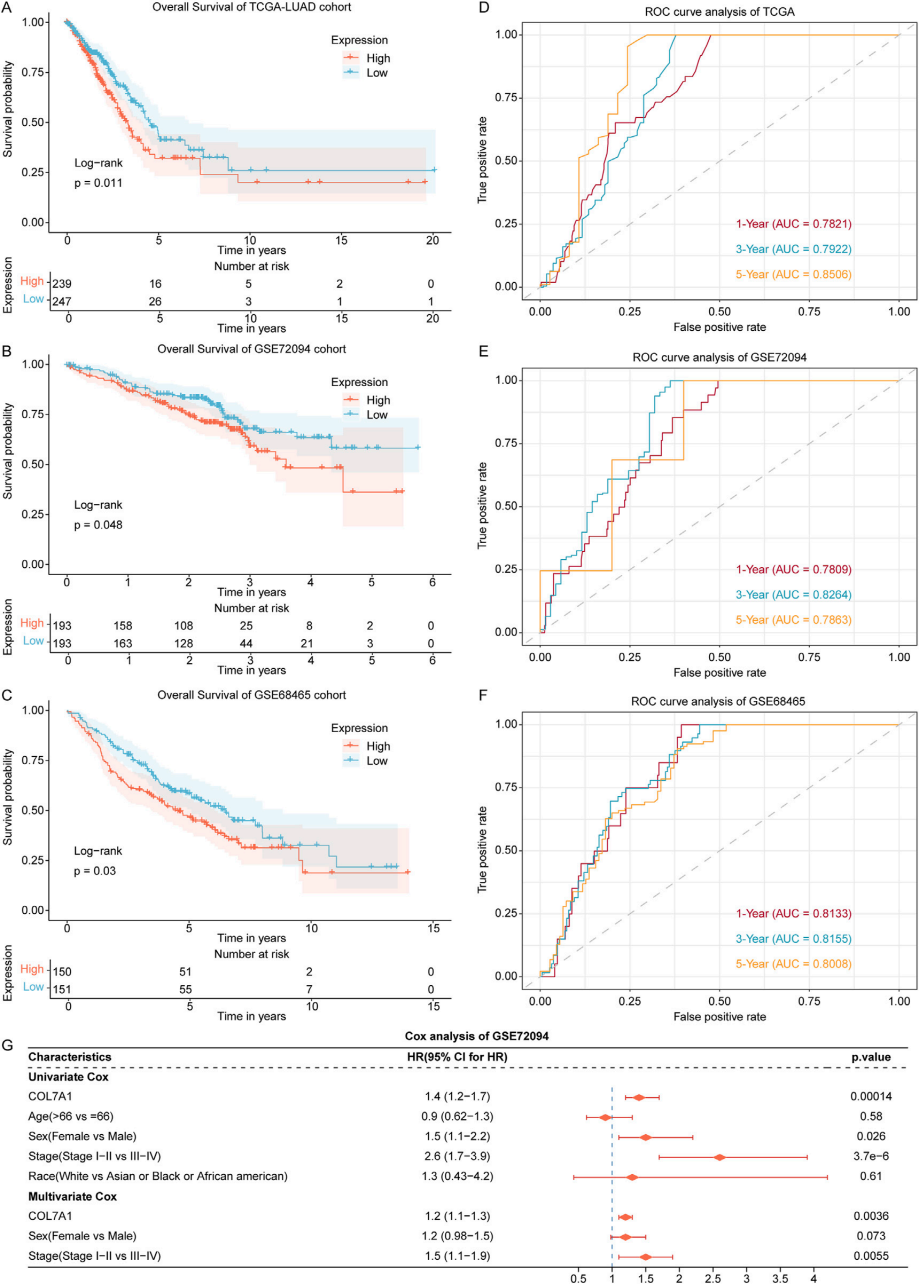
Exploration of the association of *COL7A1* expression with prognosis of LUAD patients. **(A–C)** The Kaplan-Meier curves of *COL7A1* high expression and *COL7A1* low expression groups in the LUAD patients of TCGA **(A)**, GSE72094 **(B)**, and GSE68465 **(C)** datasets. **(D–F)** Time-dependent ROC curves of the *COL7A1* expression for predicting 1-, 3-, and 5-year survival in the TCGA **(D)**, GSE72094 **(E)**, and GSE68465 **(F)** datasets. **(G)** Univariate and multivariate Cox regression analysis of *COL7A1* expression and clinical features including age, gender, clinical stage, and race.

To explore the influence of *COL7A1* expression on the prognosis of LUAD patients, we performed a survival analysis on LUAD samples from the TCGA (TCGA-LUAD), GSE72094, and GSE68465 datasets. First, we divided TCGA-LUAD, GSE72094, and GSE68465 cohorts into high-expression (COL7A1-H) and low-expression (COL7A1-L) groups according to the median *COL7A1* expression value. The results of the survival analysis showed that in all three cohorts, the COL7A1-H group had a worse OS compared to the COL7A1-L group (Figures 5A–C).

In addition, according to the time-dependent ROC analysis, the AUC of 1-year, 3-year, and 5-year survival in the three datasets were all greater than 0.78 (Figures 5D–F), indicating that *COL7A1* expression could effectively predict the prognosis of LUAD patients.

To assess the independence of *COL7A1* expression in predicting prognosis, we conducted both univariate and multivariate Cox regression analyses utilizing the TCGA-LUAD, GSE72094, and GSE68465 cohorts, incorporating four key clinical factors: age, gender, clinical stage, and race. The results of the GSE72094 cohort confirmed that *COL7A1* expression was significantly associated with OS (Figure 5E; Fig. S2A, B). Therefore, *COL7A1* expression was an independent prognostic risk factor for LUAD.

### 3.6 Exploration of the biological significance of different *COL7A1* expression groups

**FIGURE 6:**
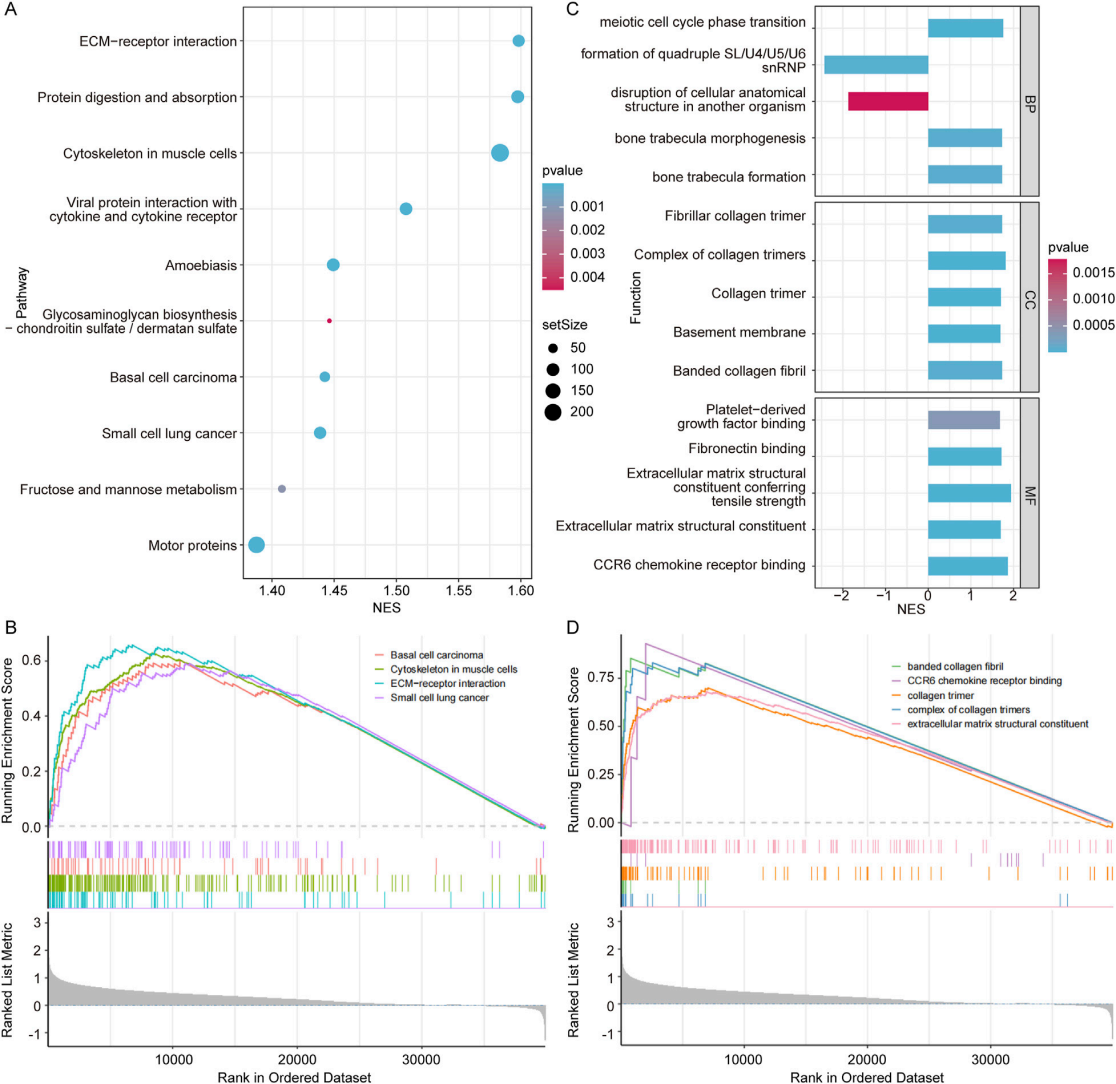
Exploration of the biological significance of different *COL7A1* expression groups. **(A)** The top 10 KEGG pathways with significant differences between the high *COL7A1* expression and low *COL7A1* expression groups. **(B)** Five important KEGG pathways were activated in the high *COL7A1* expression group based on GSEA. **(C)** The top 5 GO terms with significant differences between the high *COL7A1* expression and low *COL7A1* expression groups. **(D)** Five important GO terms were activated in the high *COL7A1* expression group based on GSEA.

To gain deeper insights into the function of *COL7A1*, we performed the GSEA on COL7A1-H and COL7A1-L groups of the TCGA-LUAD cohort. The results revealed that there were 192 pathways significantly enriched in the COL7A1-H group compared to the COL7A1-L group such as ECM-receptor interaction and protein digestion and absorption (Figure 6A; Supplementary Table S4). Notably, basal cell carcinoma, cytoskeleton in muscle cells, ECM-receptor interaction, and small cell lung cancer were significantly activated in the COL7A1-H group (Figure 6B), suggesting that high *COL7A1* expression might affect the formation and invasion of lung cancer cells in patients by activating the BM-related pathways.

In addition, the results of GO terms enrichment revealed that basement membrane pathways were significantly enriched in the COL7A1-H group compared to the COL7A1-L group (Figure 6C; Supplementary Table S4). Notably, banded collagen fibril, CCR6 chemokine receptor binding, collagen trimer, complex of collagen trimers, and extracellular matrix structural constituent were significantly activated in the COL7A1-H group (Figure 6D). These GO terms play important roles in the regulation of inflammation/immune response, tissue regeneration and repair processes.

### 3.7 Distinct immune landscape characteristics between different *COL7A1* expression groups

**FIGURE 7:**
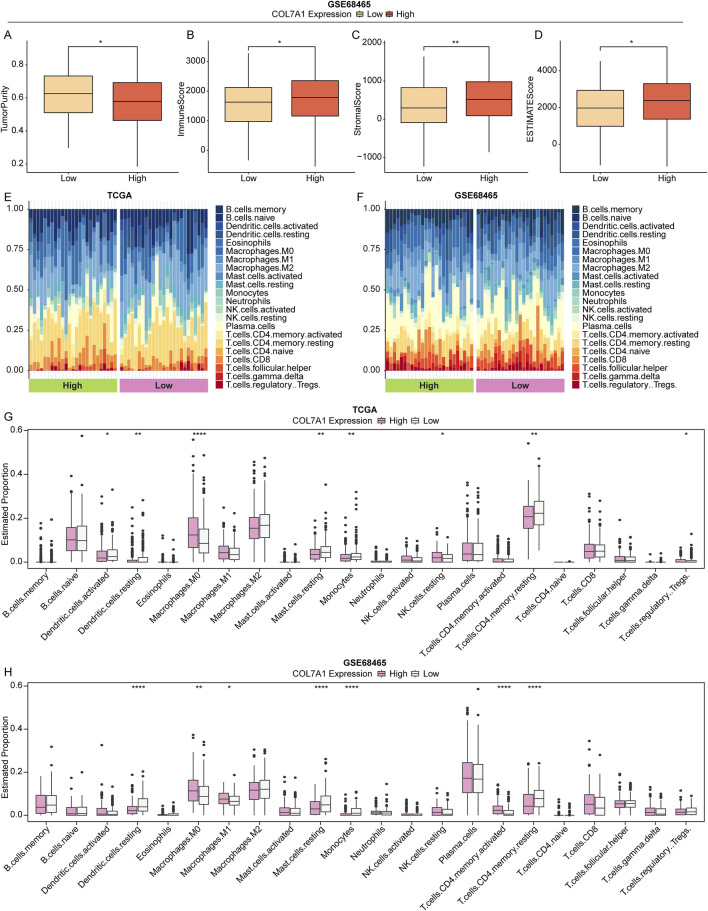
The landscape of immune cell infiltration between different groups. **(A–D)** Box plot of tumor purity **(A)**, immune **(B)**, stromal **(C)**, and ESTIMATE **(D)** scores in different *COL7A1* expression groups based on the GSE68465 dataset. **(E, F)** TStacked plot of 22 immune cell types in each sample of TCGA-LUAD **(E)** and GSE68465 **(F)** cohorts. **(G, H)** The 22 immune cell infiltration in the high *COL7A1* expression and low *COL7A1* expression groups of TCGA-LUAD **(G)** and GSE68465 **(H)** cohorts. (*p < 0.05, **p < 0.01, ***p < 0.001)

To explore the effect of *COL7A1* expression on the immune response of LUAD patients, we investigated the relationship between the *COL7A1* expression and the tumor microenvironment in LUAD patients using the ESTIMATE algorithm. Our findings indicated that, in the GSE68465 cohort, the COL7A1-H group had a lower tumor purity compared to the COL7A1-L group (Figure 7A), while the immune, stromal, and ESTIMATE scores were the opposite with significant statistical differences (Figures 7B–D). Intrestingly, we discovered a comparable trend was in the TCGA-LUAD cohort, although not statistically significant (Supplementary Figures S3A–D). These results suggested that the higher expression of *COL7A1* might trigger a stronger immune response, leading to a greater infiltration of immune cells into the tumor microenvironment.

Subsequently, we analyzed the immune landscape of patients in the TCGA-LUAD and GSE68465 cohorts to investigate the relationship between *COL7A1* expression and immune status. Using the CIBERSORT algorithm, the proportion of 22 immune cell types in the different *COL7A1* expression groups was calculated (Figures 7E, F). The results showed a significant difference in the five immune cell infiltrates between the COL7A1-H and COL7A1-L groups (Figures 7G, H). Notably the proportion of resting dendritic cells (DCs), resting mast cells, monocytes, and resting CD4 T cells was lower in the COL7A1-H group compared to the COL7A1-L group, while the opposite was observed for M0 macrophages. These observations suggested that *COL7A1* expression played a key role in the immune microenviroment of LUAD patients.

### 3.8 Drug exploitation of LUAD patients with high *COL7A1* expression

**FIGURE 8:**
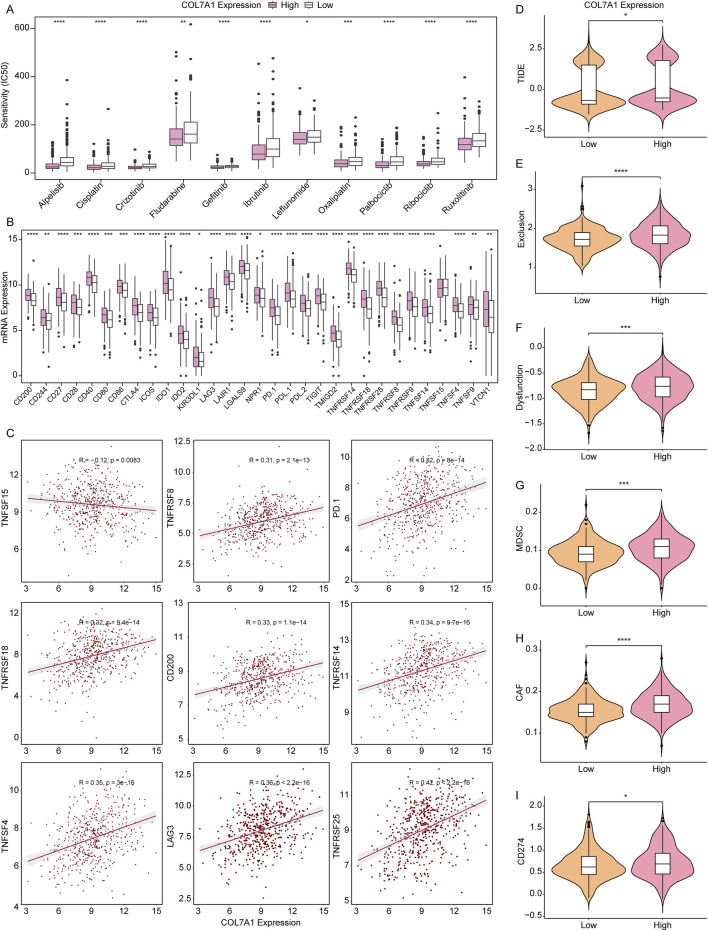
Validation of *COL7A1* expression in predicting the clinical benefit of immunotherapy. **(A)** IC50 values of the 11 drugs in the COL7A1-H and COL7A1-L groups. **(B)** The expression levels of immune checkpoint genes in different *COL7A1* expression groups. **(C)**. Correlation analysis of immune checkpoint genes and *COL7A1* expression (the data conformed to normal distribution, employing pearson correlation analysis) **(D–I)** Violin plots of TIDE **(D)**, Exclusion **(E)**, Dysfunction **(F)**, MDSC **(G)**, CAF **(H)**, and CD274 **(I)** scores in different *COL7A1* expression groups.

To establish a reference treatment protocol for LUAD patients, we delved into the association between *COL7A1* expression and the half-maximal inhibitory concentration (IC50) of various drugs. Our findings revealed that LUAD patients exhibiting high *COL7A1* expression displayed significantly lower IC50 values for 11 drugs such as alpelisib, cisplatin, and gefitinib (Figure 8A), This indicates that these patients may respond more favorably to these medications. We further examined the relationship between *COL7A1* expression and key immune checkpoints, and discovered notable differences in the expression of 30 immune checkpoint genes between the COL7A1-H and COL7A1-L groups (Figure 8B). Pearson correlation analysis highlighted a distinct negative correlation between *COL7A1* expression and TNFSF15 (*R* = −0.12, *p* = 0.0083), whereas *COL7A1* positively correlated with other drugs like TNFRSF25 (*R* = 0.42, *p* < 0.001), LAG3 (*R* = 0.36, *p* < 0.001), and TNFSF4 (*R* = 0.35, *p* < 0.001) (Figure 8C; Supplementary Figures S4, S5).

Subsequently, we delved into the clinical response of LUAD to immunotherapy utilizing the TIDE algorithm. We observed that the expression levels of several key factors—including TIDE, Exclusion, MDSC, CAF, and CD274—were conspicuously elevated in the COL7A1-H group in comparison to the COL7A1-L group (Figures 8D–I). This pattern indicates that the patients in the COL7A1-H group were more likely to escape immune surveillance and benefit less from immune checkpoint inhibition therapy.

## 4 Discussion

LUAD was the histological type of lung cancer with the highest prevalence (Thai et al., 2021). Although medical technology contributed to the treatment of LUAD, the overall prognosis of patients was still poor (Villalobos and Wistuba, 2017). BM was a key barrier to tumor cell invasion, preventing the spread of primary tumors to adjacent tissues (Mukwaya et al., 2021), and played a key role in the formation and normal function of various tissues (Pozzi et al., 2017). Therefore, a better understanding of the BM-related biomarkers in LUAD patients could contribute to the improved prognosis.

In this study, basing on multiple public cohorts and bioinformatics mining, we successfully identified *COL7A1* as a BM-related key biomarker for LUAD. It was well known that ECM served as a mechanical support/scaffold for tumor growth, cell proliferation, and migration, playing an important role in many cancers (Nallanthighal et al., 2019). The collagen type VII Alpha 1 chain (*COL7A1*) was the major structural and functional component of the ECM. *COL7A1* was the anchor fibrils of BM, mainly distributed at the dermal-epidermal junction, oral mucosa, and cervix (O'Leary et al., 2016). Germline mutations in *COL7A1* could lead to dystrophic epidermolysis bullosa, resulting in an increased risk of squamous cell carcinoma (Ortiz-Urda et al., 2005). Previous research has reported associations between COL7A1 and various cancers. For instance, *COL7A1* was highly expressed in esophageal squamous cell carcinoma and significantly associated with the depth of tumor infiltration (Kita et al., 2009). Oh et al. have demonstrated that *COL7A1* was a novel biomarker with diagnostic and therapeutic value in gastric cancer (GC), exhibiting upregulated expression in GC tissues (Oh et al., 2021). Recently, Ding et al. found that *COL7A1* expression was upregulated in pancreatic cancer (PC) and served as an independent biomarker and an influential modulator of immune infiltration (Ding et al., 2023). Intriguingly, *COL7A1* was found to be significantly over-expressed in lung squamous cell carcinoma (LUSC), where the interaction between laminin 332 and *COL7A1* might lead to the activation of the PI3K signaling pathway, ultimately contributing to squamous cell carcinoma (Song et al., 2022). Remarkably, our study revealed a similar expression pattern, with significantly higher *COL7A1* expression observed in LUAD samples. Furthermore, patients with high *COL7A1* expression exhibited a worse prognosis. The expression of *COL7A1* could effectively predict patient’s survival rates, highlighting its potential prognostic value in LUAD.

The results of functional enrichment analysis showed that *COL7A1* participated in ECM-receptor interaction and PI3K-Akt signaling pathway. The ECM-receptor interaction played an important role in the process of tumor shedding, adhesion, degradation, movement, and hyperplasia (Bao et al., 2019). The PI3K/Akt signaling pathway was a highly conserved signal transduction network in eukaryotic cells that promoted cell cycle progression and played an important role in cancer (Glaviano et al., 2023). In addition, pathways such as basal cell carcinoma and cytoskeleton in muscle cells were activated in the GOL7A1-H group. These results suggested that *COL7A1* was important to the development of LUAD cells.

In recent years, immunotherapy emerged as a remarkably popular anti-cancer therapy (Curran et al., 2010). By assessing the immune landscape in different *COL7A1* expression groups, we found that the COL7A1-H group exhibited significantly elevated immune, stromal, and ESTIMATE scores in comparison to the COL7A1-L group. This disparity likely stemmed from the fact that a higher *COL7A1* expression would provoke a stronger immune response, promoting a greater influx of immune cells and stromal cells into the tumor microenvironment. Thus, we observed immune cell infiltration status and discovered lower proportions of resting DCs, resting mast cells, and monocytes in the COL7A1-H group. DCs were the most potent antigen-presenting cells capable of physically interacting with and stimulating T lymphocytes to initiate an adaptive immune response (Thery and Amigorena, 2001). The reduced proportion of resting DCs suggested a diminished capacity of the immune system to respond to LUAD, potentially allowing cancer cells to evade immune surveillance and attack. Meanwhile, mast cells, innate immune cells resident in tissues, played a key role in inflammatory responses and tissue homeostasis, shaping the tumor microenvironment through crosstalk with other tumor-infiltrating cells (Aponte-Lopez and Munoz-Cruz, 2020). During cancer, different monocyte subpopulations contributed to tumor-promoting function (Olingy et al., 2019). This suggested that the immune system of LUAD patients in COL7A1-H group was suppressed, indicating a significant negative impact on the immune system, ultimately diminishing its ability to recognize and respond to cancer cells. This might accelerate the development of LUAD and reduce the therapeutic efficacy.

Finally, to further evaluate the potential of *COL7A1* as a therapeutic target, we evaluated the clinical response to immunotherapy in LUAD patients with different *COL7A1* expression levels. We found that the TIDE score in the COL7A1-H group was significantly higher than in the COL7A1-L group, indicating that highly *COL7A1* expressed patients were more likely to evade the immune system and benefit less from immune checkpoint suppression therapy. Nevertheless, to the best of our knowledge, rare studies have focused on therapeutic approaches targeting *COL7A1*, neither *COL7A1* inhibitors nor gene therapies. It reminds us that there are still great research gaps underlying *COL7A1*, especially involving its potential as a therapeutic target. More details involving immunotherapy in LUAD patients with different *COL7A1* expressions deserved further investigation. In our future work, drug development targeting *COL7A1* would be a meaningful aspect in improving the clinical outcomes of LUAD patients, such as *COL7A1* inhibitors and gene therapy targeting *COL7A1*.

In this work, despite discovery of preliminary potential roles of *COL7A1* in LUAD via multiple public cohorts and *in vitro* validation, there are still some limitations. Firstly, although our present work has included as many public datasets as possible to avoid potential data bias, we have to recognize the potential limitation of retrospective data and distinct sample characteristics. Moreover, there was a lack of clinical cohort exploration, thus further investigation and validation in clinical trials should be performed in the future work. On the other hand, our present study has focused on a preliminary role of *COL7A1*, and more deepening investigation is necessary. In the near future, the purpose of our next study is to explore the impacts of *COL7A1* on the malignant progression of LUAD (employing wet-lab experimental tools), based on which the further clinical application of *COL7A1* in predictive marker, drug development and targeting treatment would be quite helpful in improving patients’ quality of life.

## 5 Conclusion

Our study successfully identified *COL7A1* as a BM-related key biomarker holding promising potential in predicting the prognosis of LUAD. Patients with high *COL7A1* expression had a worse prognosis and greater immune escape ability than those *COL7A1* lowly expressed patients. Functional enrichment analysis revealed that *COL7A1* was involved in the biological pathways relating to cell proliferation and inflammation. This study offered fresh insights into the mechanistic understanding of BM in LUAD, especially involving *COL7A1*.

## Data Availability

The original contributions presented in the study are included in the article/Supplementary Material, further inquiries can be directed to the corresponding authors.
