# Supplementary figures and images for: COL7A1 indicates crucial potential as a basal membrane-related prognostic biomarker and therapeutic target in lung adenocarcinoma
Source: Front Pharmacol. 2025 Feb 14;16:1543193. doi: 10.3389/fphar.2025.1543193 (PMC11868062; doi:10.3389/fphar.2025.1543193)

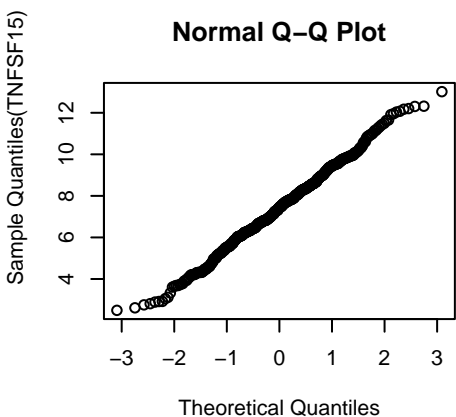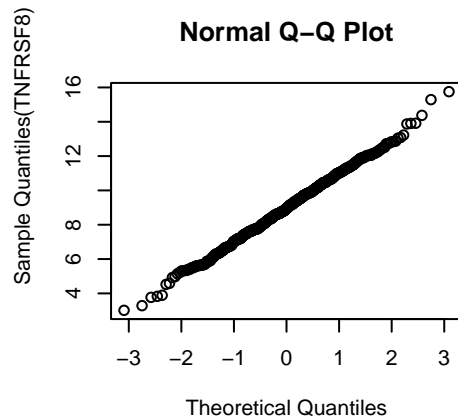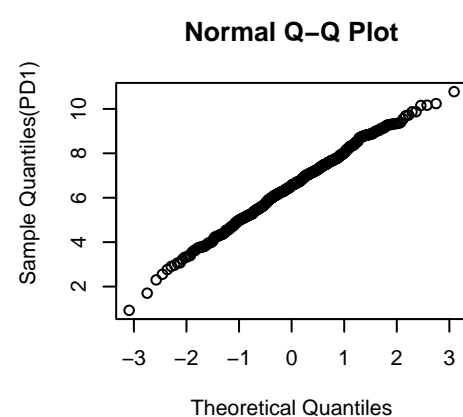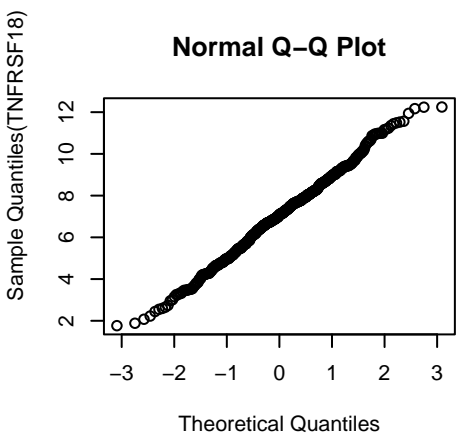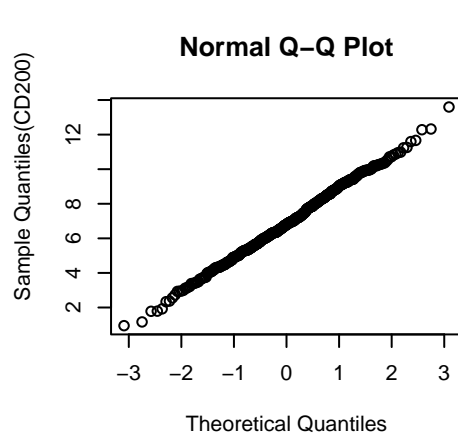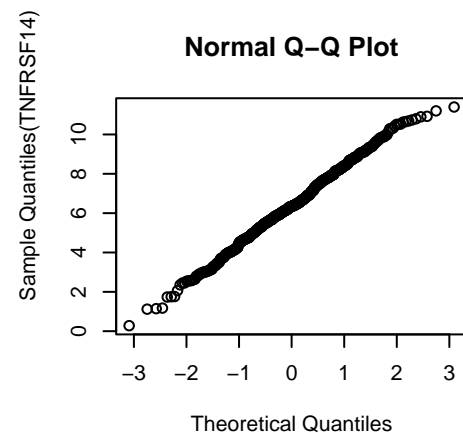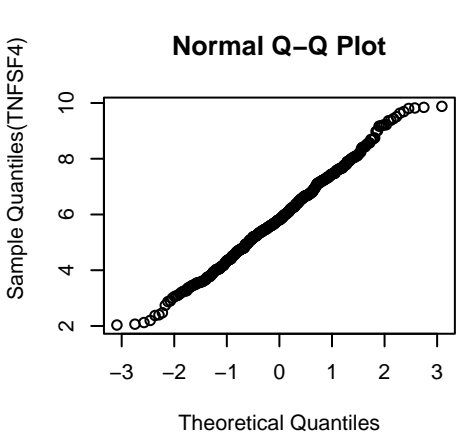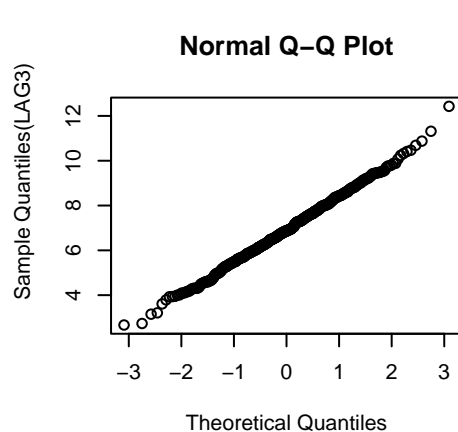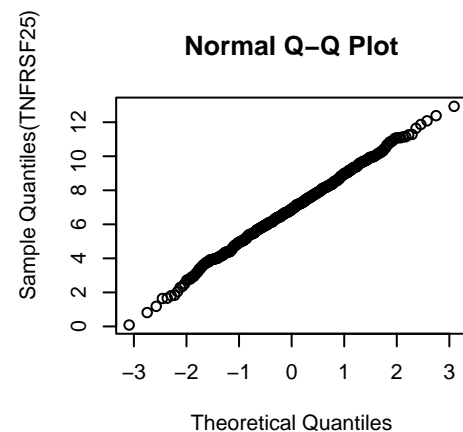

Supplement: Supplementary file 1 [file Image5.pdf]

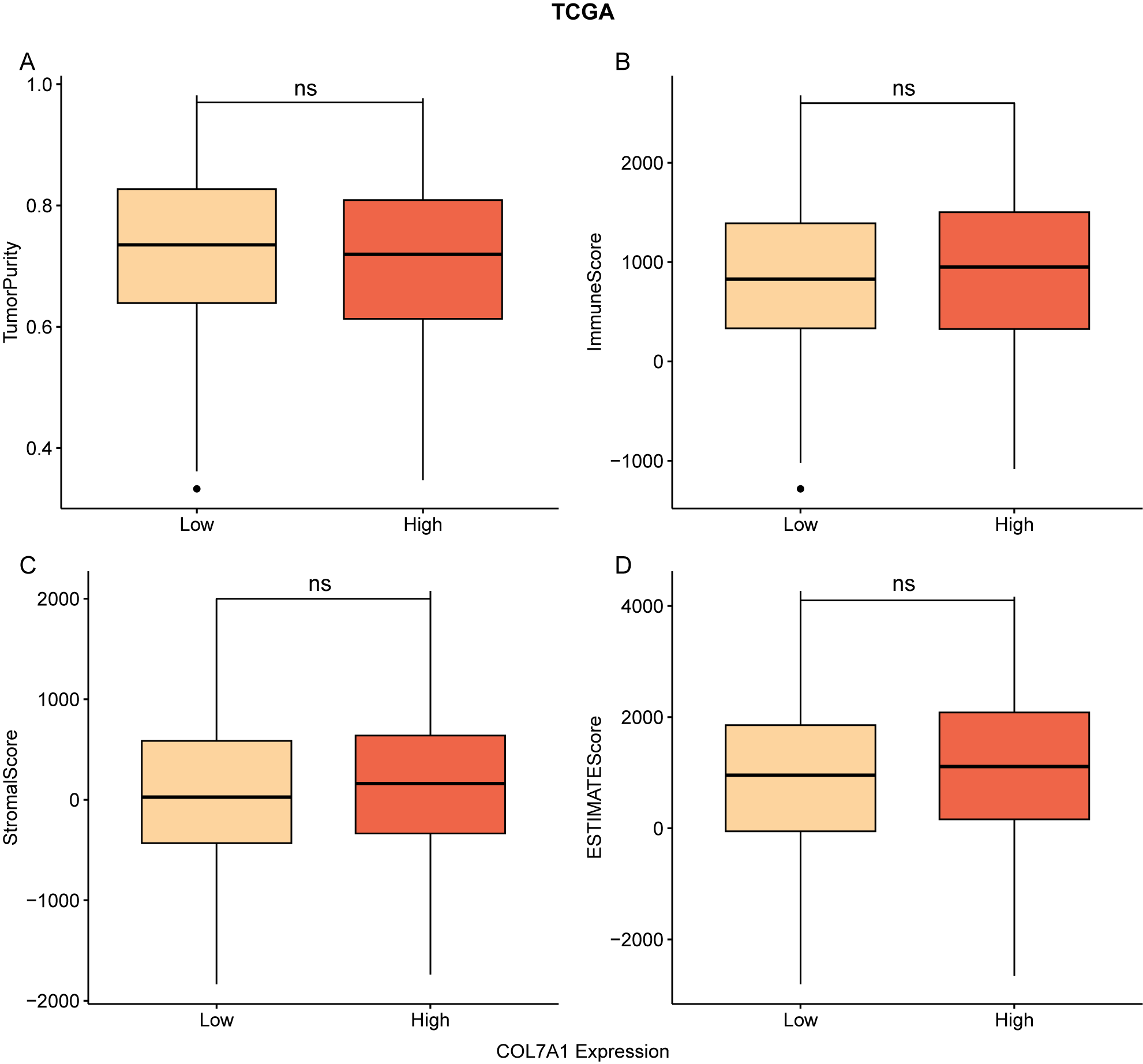

Supplement: Supplementary file 3 [file Image3.tif]

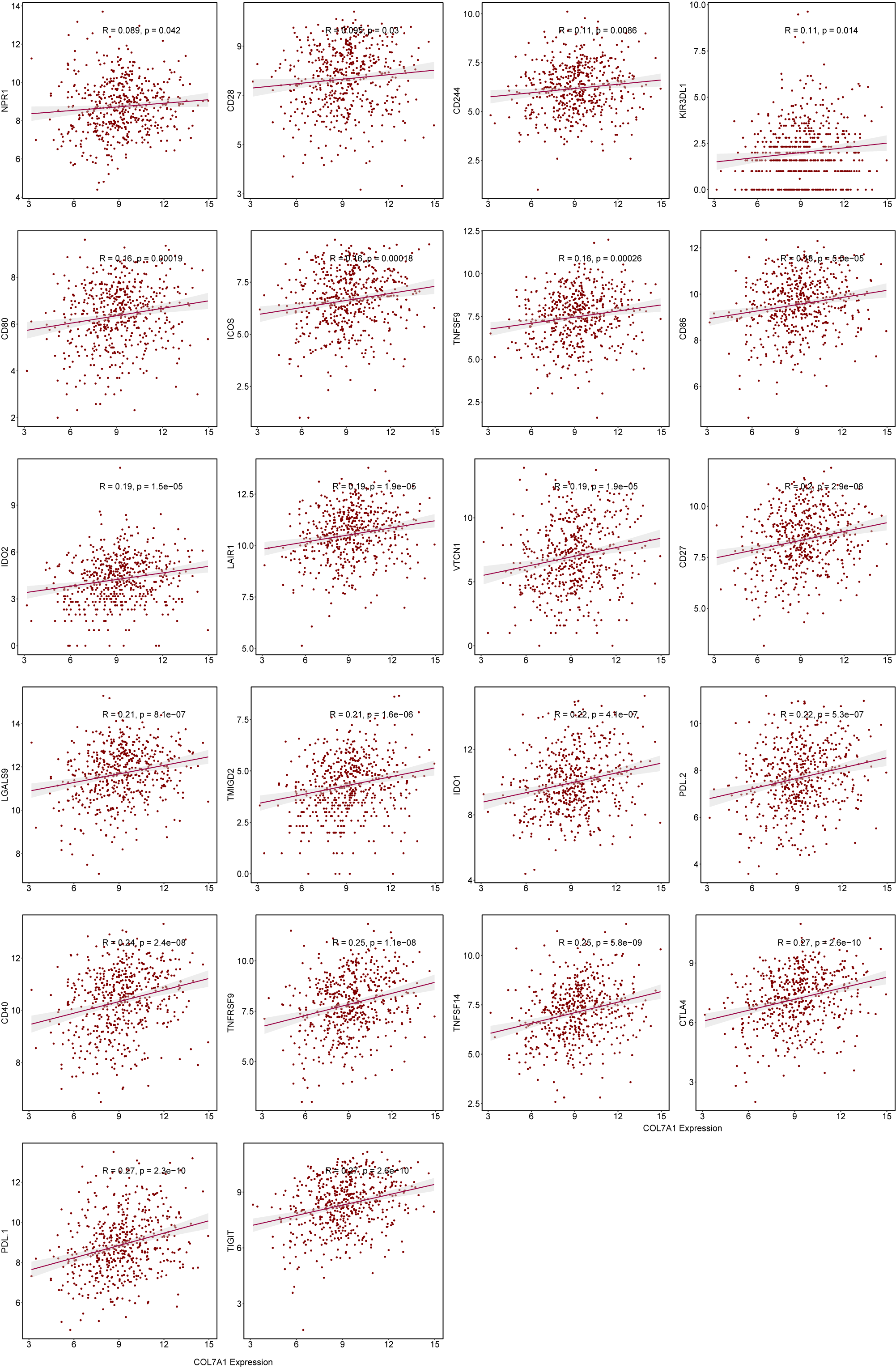

Supplement: Supplementary file 4 [file Image4.tif]

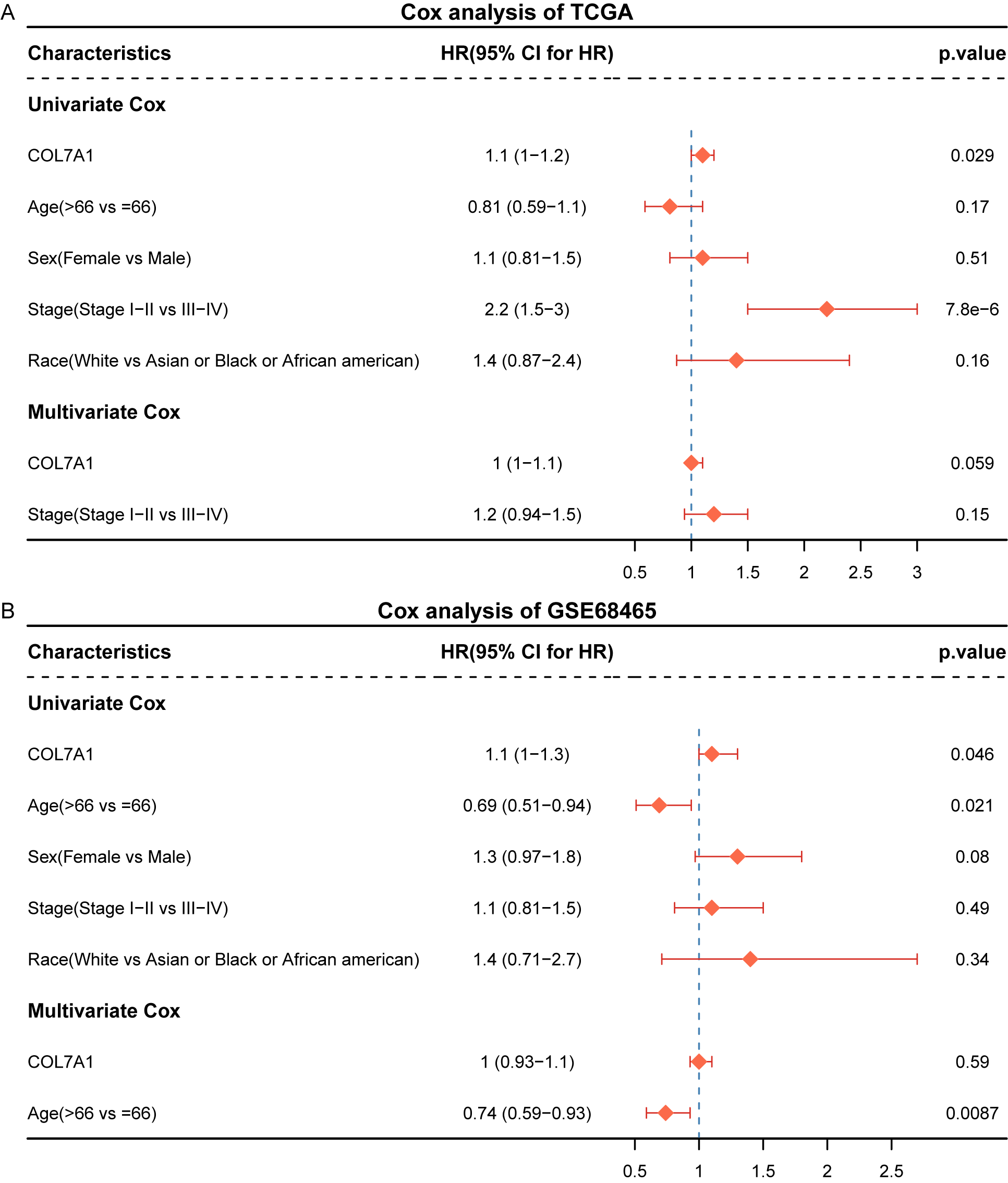

Supplement: Supplementary file 5 [file Image2.tif]

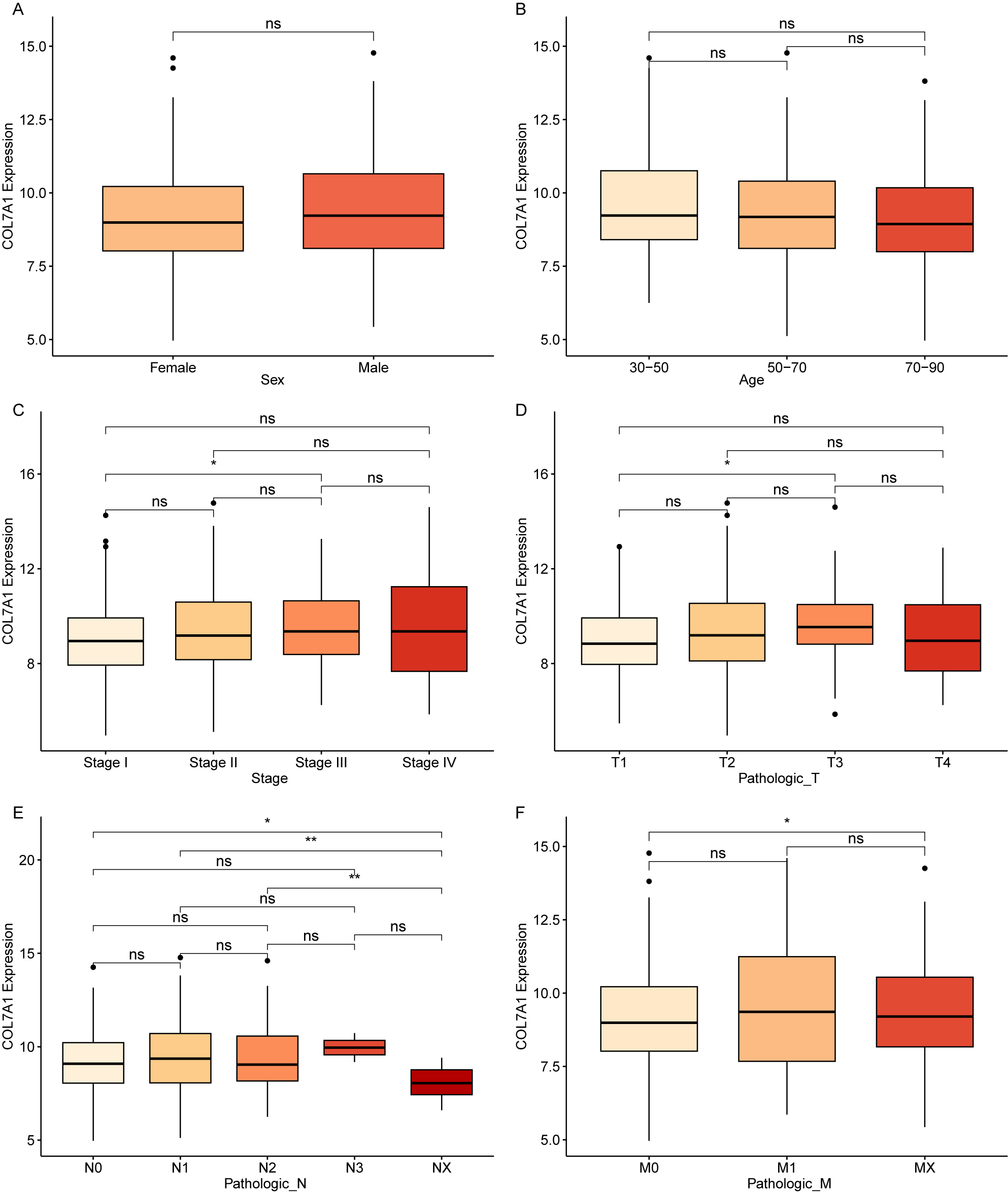

Supplement: Supplementary file 6 [file Image1.tif]
